# Supplementary figures and images for: Anti-inflammatory effects of eupatilin on Helicobacter pylori CagA-induced gastric inflammation
Source: PLoS One. 2024 Nov 5;19(11):e0313251. doi: 10.1371/journal.pone.0313251 (PMC11537371; doi:10.1371/journal.pone.0313251)

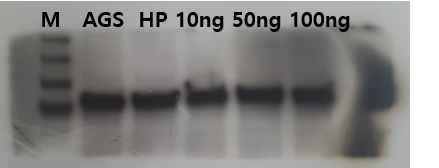

Supplement: S1 Fig — (TIF) [file pone.0313251.s001.tif]

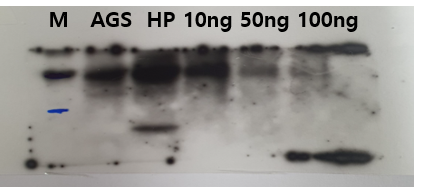

Supplement: S2 Fig — (TIF) [file pone.0313251.s002.tif]

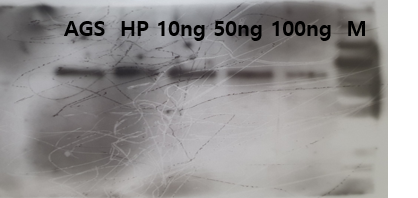

Supplement: S3 Fig — (TIF) [file pone.0313251.s003.tif]

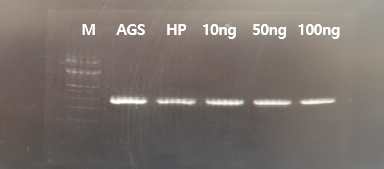

Supplement: S4 Fig — (TIF) [file pone.0313251.s004.tif]

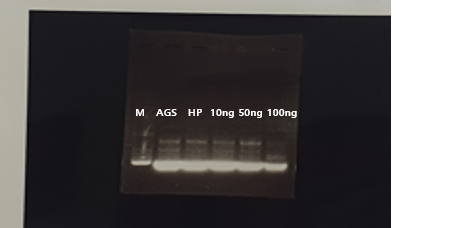

Supplement: S5 Fig — (TIF) [file pone.0313251.s005.tif]

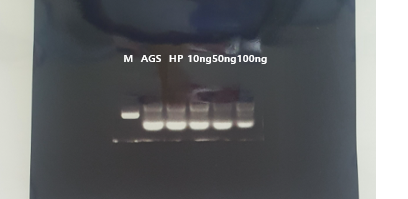

Supplement: S6 Fig — (TIF) [file pone.0313251.s006.tif]

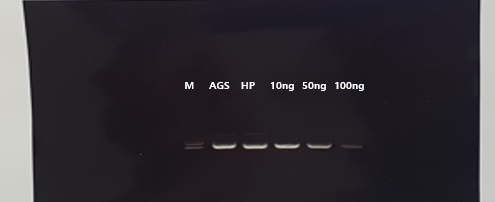

Supplement: S7 Fig — (TIF) [file pone.0313251.s007.tif]

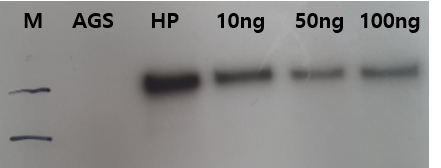

Supplement: S8 Fig — (TIF) [file pone.0313251.s008.tif]

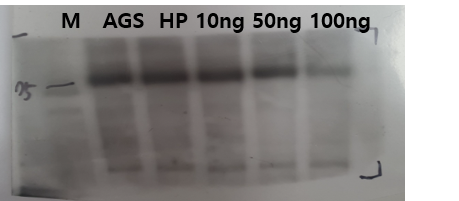

Supplement: S9 Fig — (TIF) [file pone.0313251.s009.tif]

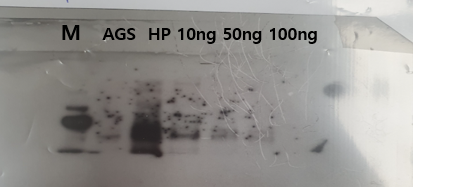

Supplement: S10 Fig — (TIF) [file pone.0313251.s010.tif]
