# Supplementary material for: Anti-inflammatory effects of eupatilin on Helicobacter pylori CagA-induced gastric inflammation
Source: PLoS One. 2024 Nov 5;19(11):e0313251. doi: 10.1371/journal.pone.0313251 (PMC11537371; doi:10.1371/journal.pone.0313251)
Supplement: S11 Fig — (PPTX) [file pone.0313251.s011.pptx]

## Slide 1
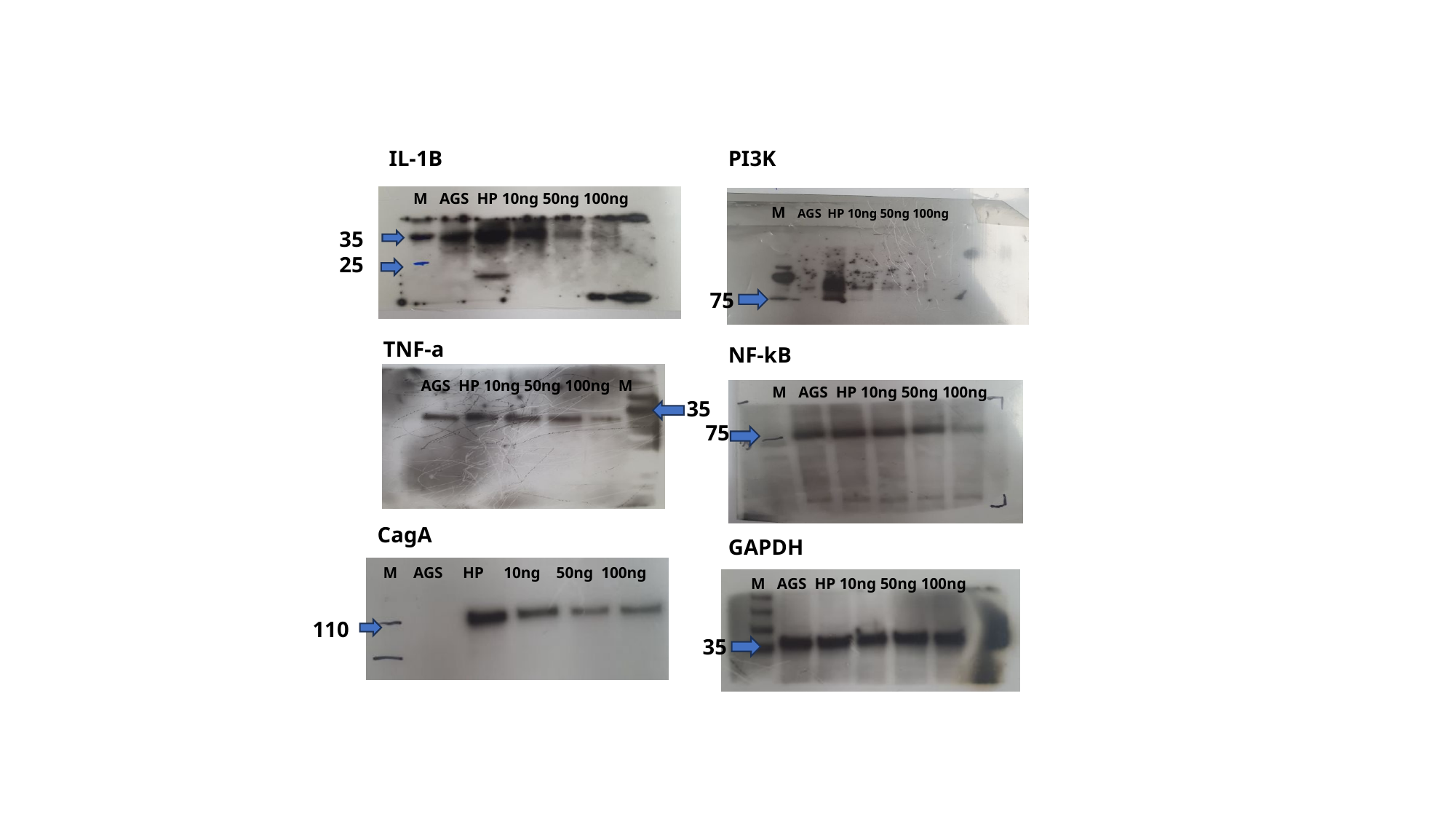

IL-1B
PI3K
M AGS HP 10ng 50ng 100ng
M AGS HP 10ng 50ng 100ng
35
25
75
TNF-a
NF-kB
 AGS HP 10ng 50ng 100ng M
M AGS HP 10ng 50ng 100ng
35
75
CagA
GAPDH
M AGS HP 10ng 50ng 100ng
M AGS HP 10ng 50ng 100ng
110
35
