# Supplementary material for: Anti-inflammatory effects of eupatilin on Helicobacter pylori CagA-induced gastric inflammation
Source: PLoS One. 2024 Nov 5;19(11):e0313251. doi: 10.1371/journal.pone.0313251 (PMC11537371; doi:10.1371/journal.pone.0313251)
Supplement: Attachment — Submitted filename: REVISION_PlosOne.docx [file pone.0313251.s013.docx]

The authors investigate the anti-inflammatory effects of eupatilin in gastric epithelial cells infected with Helicobacter pylori (H. pylori). The results show that eupatilin inhibits the translocation of CagA and suppresses the PI3K/NF-κB signaling pathway, thereby reducing the expression of pro-inflammatory cytokines in a dose-dependent manner. This anti-inflammatory effect suggests that eupatilin may play a protective role against H. pylori-induced gastritis, highlighting its potential as a therapeutic alternative in the context of increasing antibiotic resistance and possible gastric dysbiosis post-treatment. However, the study presents certain limitations that need to be addressed to improve the work.

Major:

1. To confirm the anti-inflammatory effects of eupatilin, the authors should conduct blocking or inhibition studies using siRNAs or specific blockers (e.g., PI3K/NF-κB) to corroborate if it indeed inhibits these molecular pathways.

2. Related to the previous point, another option would be to compare the results with those obtained using already established and safe therapies.

3. Despite strong in vitro evidence, the exact mechanisms by which eupatilin acts in vivo remain unclear. The interaction of H. pylori with the host involves complex host-environment factors, which are not fully replicated in in vitro studies. This point should be included as a limitation if the authors do not have in vivo study results.

4. While the study demonstrates the anti-inflammatory properties of eupatilin, its direct anti-tumor effects were not investigated. Future research should explore whether eupatilin has potential benefits in preventing or treating gastric cancer.

Minor:

1. In the different figures, HP should be defined in the figure legends.

2. Review Figure 4: Figures 4A and 4B are western blots, but the text describes something else.

3. Sometimes the text references Figs 4C–G and other times Fig 5A and 5B. Ensure all references have the same style.
