# Supplementary material for: Anti-inflammatory effects of eupatilin on Helicobacter pylori CagA-induced gastric inflammation
Source: PLoS One. 2024 Nov 5;19(11):e0313251. doi: 10.1371/journal.pone.0313251 (PMC11537371; doi:10.1371/journal.pone.0313251)
Supplement: Attachment — Submitted filename: Response to reviewers final.docx [file pone.0313251.s014.docx]

**We truly appreciate your kind review and comments. We tried our best to revise our manuscript according to your recommendations and highlighted the revised parts in red for your convenience of re-reviewing. Details of our response to your comments are described below. We hope our manuscript has been improved accordingly and is now suitable for publication in your journal.**

Major:

1. To confirm the anti-inflammatory effects of eupatilin, the authors should conduct blocking or inhibition studies using siRNAs or specific blockers (e.g., PI3K/NF-κB) to corroborate if it indeed inhibits these molecular pathways.

2. Related to the previous point, another option would be to compare the results with those obtained using already established and safe therapies.

**-> Answer to questions 1 and 2:**

**Thank you for your kind review and advices. We apologize for not being able to provide additional blocking/inhibition or comparison studies because of our current laboratory circumstances. Although your comments are meaningful and we agree with your suggestions, practical issues prevent us from following them since doing so would mean redoing everything from the beginning.**

**Despite these limitations, we believe our research provides supporting evidence of the anti-inflammatory effects of eupatilin by suppressing the expression of PI3K/NF-κB. We initially focused on the inhibitory effect of eupatilin on *H. pylori* CagA (initial step of anti-inflammatory process) rather than on its direct activity on PI3K/NF-κB pathways. It is a well-known hypothesis that *H. pylori* CagA activates the PI3K/NF-κB signaling pathway. In our research, we determined that eupatilin treatment dose-dependently inhibited CagA expression and thus suppressed CagA-induced PI3K/NF-κB pathways.**

**We additionally described this issue as a limitation in the discussion section as follows:**

Moreover, we did not confirm the direct effect of eupatilin on PI3K/NF-κB signaling. We initially focused on the inhibitory effect of eupatilin on *H. pylori* cagA, a factor known to activate the PI3K/NF-κB signaling pathway, rather than on its direct activity on PI3K/NF-κB pathways. We determined that eupatilin treatment dose-dependently inhibited CagA expression, which could sufficiently suppress CagA-induced PI3K/ NF-κB pathways.

**We hope our explanation is sufficiently understandable, and we look forward to hearing a positive response. Thank you for your kind comments, and we will plan our next research based on your suggestions.**

3. Despite strong in vitro evidence, the exact mechanisms by which eupatilin acts in vivo remain unclear. The interaction of H. pylori with the host involves complex host-environment factors, which are not fully replicated in in vitro studies. This point should be included as a limitation if the authors do not have in vivo study results.

**-> Thank you for comment. As you suggested, we have added this point as a limitation in the revised manuscript as follows:**

**Despite convincing *in vitro* evidence of the anti-inflammatory effects of eupatilin on *H. pylori* CagA-infected gastric epithelial cells, the exact mechanisms of action of eupatilin *in vivo* remain unclear. The interaction of *H. pylori* with the host involves complex host–environment factors that could not be fully replicated in *in vitro* studies. Thus, further *in vivo* studies are needed to determine the safe and effective dose of eupatilin to exert anti-inflammatory effects against *H. pylori*-induced gastritis.**

4. While the study demonstrates the anti-inflammatory properties of eupatilin, its direct anti-tumor effects were not investigated. Future research should explore whether eupatilin has potential benefits in preventing or treating gastric cancer.

**-> Thank you for your comment. As you suggested, we have added this point as a future scope for study in the revised manuscript as follows:**

**Future research should be conducted to reveal other anti-inflammatory pathways of eupatilin and identify whether it exerts a direct anti-tumor activity in preventing or treating gastric cancer.**

Minor:

1. In the different figures, HP should be defined in the figure legends.

**-> Thank you for your detailed comment. We have added the definition of HP (HP = *Helicobacter pylori*) in each figure legend.**

2. Review Figure 4: Figures 4A and 4B are western blots, but the text describes something else.

**-> We are sorry but we described the methods appropriately. Figure 4A shows the results of western blot (black bands), and Figure 4B shows the results of RT-PCR (white bands).**

3. Sometimes the text references Figs 4C–G and other times Fig 5A and 5B. Ensure all references have the same style.

**-> Thank you for your detailed comment. We have revised “4C-G” to “4C, D, E, F, and G.”**
