# Supplementary material for: Anti-inflammatory effects of eupatilin on Helicobacter pylori CagA-induced gastric inflammation
Source: PLoS One. 2024 Nov 5;19(11):e0313251. doi: 10.1371/journal.pone.0313251 (PMC11537371; doi:10.1371/journal.pone.0313251)
Supplement: Attachment — Submitted filename: Response to reviewers final2.docx [file pone.0313251.s015.docx]

**We truly appreciate your kind review and comments. We tried our best to revise our manuscript according to your recommendations and highlighted the revised parts in red for your convenience of re-reviewing. Details of our response to your comments are described below. We hope our manuscript has been improved accordingly and is now suitable for publication in your journal.**

1. Please elaborate more on why a gastric carcinoma cell line was used in this study instead of a normal gastric cell line, given that it already shows a heightened level of inflammation before incubation with CagA. If it is not possible to perform a control experiment using a normal cell line, please elaborate more on this issue in the discussion section by providing enough justification and references to the below part:
“Nevertheless, H. pylori-infected AGS cells can serve as a model for normal gastric epithelial cells exposed to H. pylori, given that AGS cells have been extensively employed in experiments on the response to H. pylori.”

**-> Thank you for your comment. We additionally described this issue as a limitation in the discussion section as follows:**

We employed AGS human gastric carcinoma cell line instead of using normal gastric cell line, though AGS cells already could possess a heightened level of inflammation before *H. pylori* infection. Nevertheless, AGS cells have strong cell viability and have been extensively employed to understand the consequences of infection on epithelial cells, particularly in experiments on the response to *H. pylori* [16]. Therefore, we thought that AGS cells can serve as an alternative model for normal gastric epithelial cell in this research.

(Reference 16. Matsunaga S, Nishiumi S, Tagawa R, Yoshida M. Alterations in metabolic pathways in gastric epithelial cells infected with Helicobacter pylori. Microb Pathog 2018;124:122-129.)

2. The reference list is quite short for such a well-established disease model. Specifically, the materials and methods section does not include any references. Please add appropriate references for each of the methodologies used and for the primer sequences used in the PCR experiment.

**-> Thank you for your comment. We added references for methodologies used in the PCR experiment in part of ‘Analysis of mRNA expression’. (References 13,14,15)**

3. Please add a reference for the hummingbird morphological changes expected after translocation, as it seems like a crucial verification point of translocation in this model.

**-> Thank you for your comment. We added references for the hummingbird morphological changes expected after CagA translocation in part of ‘Effects of eupatilin on CagA translocation into AGS cells and CagA-induced morphologic changes’ (References 16,17)**

4. Figure 1 is missing the statistical p-values on the bar graph.

**-> Thank you for your comment. Since the purpose of cell viability test is not to compare the statistical value but to determine the treatment concentration (more than 80% of cell viability), we thought that it was not necessary to obtain p values. We referred to the research below to test for cell viability.**

Yeon MJ, Lee MH, Kim DH, et al. Anti-inflammatory effects of Kaempferol on Helicobacter pylori-induced inflammation. 2019;83166-173.

Choi EJ, Lee [S](https://scholar.google.co.kr/citations?user=m1YgQIQAAAAJ&hl=ko&oi=sra), Char JR, et al. Eupatilin inhibits lipopolysaccharide-induced expression of inflammatory mediators in macrophages. Life Sci. 2011;88:1121-1126.

5. Western blot figures are missing the ladder, specifically the exact weight of the isolated bands for all the assessed markers.

**-> Thank you for your detailed comment. We loaded samples onto one gel and cut the membrane for each molecular weight. We are sorry for not showing the ladder, but we indicated markers with the molecular weight in PPT file ‘Revision (marker)’. We can say that our experiments are reliable, and we hope our explanation is sufficiently understandable. We look forward to hearing a positive response.**
