# Supplementary material for: Anti-inflammatory effects of eupatilin on Helicobacter pylori CagA-induced gastric inflammation
Source: PLoS One. 2024 Nov 5;19(11):e0313251. doi: 10.1371/journal.pone.0313251 (PMC11537371; doi:10.1371/journal.pone.0313251)
Supplement: Attachment — Submitted filename: Response to reviewers final4.docx [file pone.0313251.s016.docx]

**We appreciate your kind comment, and we added p-values in the revised figure 1. Additionally, we submitted a supporting file including the values of mean, standard error of the mean, and the *p*-value which was used to build graphs. And we also added the method to analyze the protein and RNA bands into graphs in the manuscript. We hope our manuscript has been improved accordingly and is now suitable for publication in your journal.**

1. Thank you for your response. While I understand your rationale for not including p-values, I would strongly recommend reconsidering this approach. Even if the primary goal is to identify treatment concentrations that maintain cell viability above 80%, statistical analysis is still essential to ensure that the observed effects are significant and not due to random variation. Including p-values will add robustness to your conclusions and allow for a more scientifically rigorous interpretation of the results.

Statistical significance can help validate that the differences you observe between control and treated groups are meaningful, and it provides a clearer understanding of the treatment's impact. This is a standard expectation in most scientific publications, and adding this analysis will undoubtedly strengthen your manuscript.

I urge you to include appropriate statistical tests for your cell viability data to meet the required standards and ensure your results are well-supported.

Thank you for your attention to this matter.

**-> Thank you for your comment, and we added p-values in the revised figure (showing cell viability) according to your recommendation.**
